# Immunopathological properties of the *Campylobacter jejuni* flagellins and the adhesin CadF as assessed in a clinical murine infection model

**DOI:** 10.1186/s13099-019-0306-9

**Published:** 2019-05-17

**Authors:** Anna-Maria Schmidt, Ulrike Escher, Soraya Mousavi, Nicole Tegtmeyer, Manja Boehm, Steffen Backert, Stefan Bereswill, Markus M. Heimesaat

**Affiliations:** 1 Department of Microbiology, Institute of Microbiology, Infectious Diseases and Immunology, Charité-University Medicine Berlin, corporate member of Freie Universität Berlin, Humboldt-Universität zu Berlin, and Berlin Institute of Health, CC5, Campus Benjamin Franklin, FEM, Garystr. 5, 14195 Berlin, Germany; 2 Institute for Microbiology, Department of Biology, Friedrich Alexander University Erlangen/Nuremberg, Erlangen, Germany

**Keywords:** *Campylobacter jejuni*, FlaA/B, CadF, Flagellin, IL-10^−/−^ mice, Secondary abiotic (gnotobiotic) mice, Pro-inflammatory immune responses, Host–pathogen-interaction, Bacterial translocation, Intestinal immunopathology, Extra-intestinal immune responses, Systemic immune responses

## Abstract

**Background:**

*Campylobacter jejuni* infections constitute serious threats to human health with increasing prevalences worldwide. Our knowledge regarding the molecular mechanisms underlying host–pathogen interactions is still limited. Our group has established a clinical *C. jejuni* infection model based on abiotic IL-10^−/−^ mice mimicking key features of human campylobacteriosis. In order to further validate this model for unraveling pathogen-host interactions mounting in acute disease, we here surveyed the immunopathological features of the important *C. jejuni* virulence factors FlaA and FlaB and the major adhesin CadF (*Campylobacter* adhesin to fibronectin), which play a role in bacterial motility, protein secretion and adhesion, respectively.

**Methods and results:**

Therefore, abiotic IL-10^−/−^ mice were perorally infected with *C. jejuni* strain 81-176 (WT) or with its isogenic *flaA/B* (Δ*flaA/B*) or *cadF* (Δ*cadF*) deletion mutants. Cultural analyses revealed that WT and Δ*cadF* but not Δ*flaA/B* bacteria stably colonized the stomach, duodenum and ileum, whereas all three strains were present in the colon at comparably high loads on day 6 post-infection. Remarkably, despite high colonic colonization densities, murine infection with the Δ*flaA/B* strain did not result in overt campylobacteriosis, whereas mice infected with Δ*cadF* or WT were suffering from acute enterocolitis at day 6 post-infection. These symptoms coincided with pronounced pro-inflammatory immune responses, not only in the intestinal tract, but also in other organs such as the liver and kidneys and were accompanied with systemic inflammatory responses as indicated by increased serum MCP-1 concentrations following *C. jejuni* Δ*cadF* or WT, but not Δ*flaA/B* strain infection.

**Conclusion:**

For the first time, our observations revealed that the *C. jejuni* flagellins A/B, but not adhesion mediated by CadF, are essential for inducing murine campylobacteriosis. Furthermore, the secondary abiotic IL-10^−/−^ infection model has been proven suitable not only for detailed investigations of immunological aspects of campylobacteriosis, but also for differential analyses of the roles of distinct *C. jejuni* virulence factors in induction and progression of disease.

**Electronic supplementary material:**

The online version of this article (10.1186/s13099-019-0306-9) contains supplementary material, which is available to authorized users.

## Background

*Campylobacter jejuni* are spiral-shaped, highly motile, Gram-negative bacteria that frequenly asymptomatically colonize birds, including poultry. In humans the bacteria cause campylobacteriosis, the most prevalent cause for enteric bacterial infections [1–4]. Human *C. jejuni* infections are predominantly caused by consumption of contaminated animal products and surface water [5]. Campylobacteriosis is accompanied with clinical manifestations such as abdominal pain, fever, and watery or bloody diarrhea that are mostly self-limiting [1, 6, 7]. In a minority of cases, severe post-infectious sequelae such as Guillain-Barré syndrome or reactive arthritis can occur [7, 8].

The exact molecular mechanisms underlying the development of acute and invasive enterocolitis that is typical for campylobacteriosis are unclear, but the immunopathological nature of the disease has been recognized for decades [6]. We and others have shown that *C. jejuni* interact with pattern recognition receptors such as Toll-like receptor 4 (TLR-4) [9] and nucleotide-oligomerization-domain-2 (Nod2) [10, 11], and interfere with signaling pathways dependent on MAPK/ERK (mitogen-activated protein kinases/extracellular signal-regulated kinases) and NF-κB (nuclear factor kappa-light-chain-enhancer of activated B cells) [12]. Activation of those signaling cascades induces the expression of a variety of immune response genes [13, 14]. As a result, an inflammation response is triggered, characterized by the recruitment of immune cells to the site of infection and up-regulation of cytokine production [14].

As a prerequisite for induced immunopathology, *C. jejuni* needs to adhere to and invade into epithelial host cells. Amongst a number of other factors, the flagellar filaments consisting of FlaA and FlaB, and the major adhesin CadF (*Campylobacter* adhesin to fibronectin) are considered to be major players in these processes [15]. To adhere to intestinal host cells, the bacteria need to cross the overlying mucus layer by flagella-generated motility [16]. Moreover, the flagellum can secrete molecules that promote *C. jejuni* adhesion to and invasion into host cells [17–20]. The adhesin CadF permits host cell adhesion by binding to the extracellular matrix protein fibronectin, which enables the interaction with integrin receptors and results in bacterial internalization into host cells [19, 21, 22].

The dependence of adherence and invasion on flagella has been demonstrated in vitro and in vivo by gene knockout experiments [23, 24]. It was also shown that knockout of *cadF* resulted in reduced adhesion and invasion of *C. jejuni* into host cells in vitro [21, 25] and abolished colonisation in the chicken host [26]. Both the flagellum and CadF also activate a signaling cascade in cultured INT-407 cells and other cell lines that results in the activation of the small Rho GTPase Rac1, which in turn leads to actin and/or microtubule rearrangements that trigger internalization of *C. jejuni* [27].

In order to study pathogenesis, treatment and prophylaxis of campylobacteriosis in vertebrate hosts in more detail, we have established a murine *C. jejuni* infection model based on secondary abiotic IL-10^−/−^ mice that not only allows for investigation of colonisation properties, but also reproducibly displays clinical symptoms resembling those of the compromized infected human host [28–30]. Applying this clinical infection model we have recently shown, for instance, that *C. jejuni* lipooligosaccharide (LOS) is essential for the induction of campylobacteriosis and this pathogen surface molecule thus represent an important *C. jejuni* pathogenicity factor [28, 31, 32].

To further validate this murine infection model for the study of *C. jejuni* virulence factors for induction and progression of acute disease, we here addressed whether bacterial flagella and the major adhesin CadF are pivotal prerequisites for inducing enteric disease in the murine host. To this aim, we infected secondary abiotic IL-10^−*/*−^ mice with *C. jejuni* strain 81-176, its isogenic non-motile mutant ∆*flaA/B* and its CadF-deficient ∆*cadF* mutant. The colonization capacities of these isogenic strains were compared, while clinical outcome as well as intestinal, extraintestinal and systemic immunopathologocal responses were monitored and bacterial translocation to extra-intestinal organs was determined.

## Results

### The impact of *C. jejuni* motility and adhesion to intestinal colonization following peroral infection of secondary abiotic IL-10^−/−^ mice

**Fig. 1:**
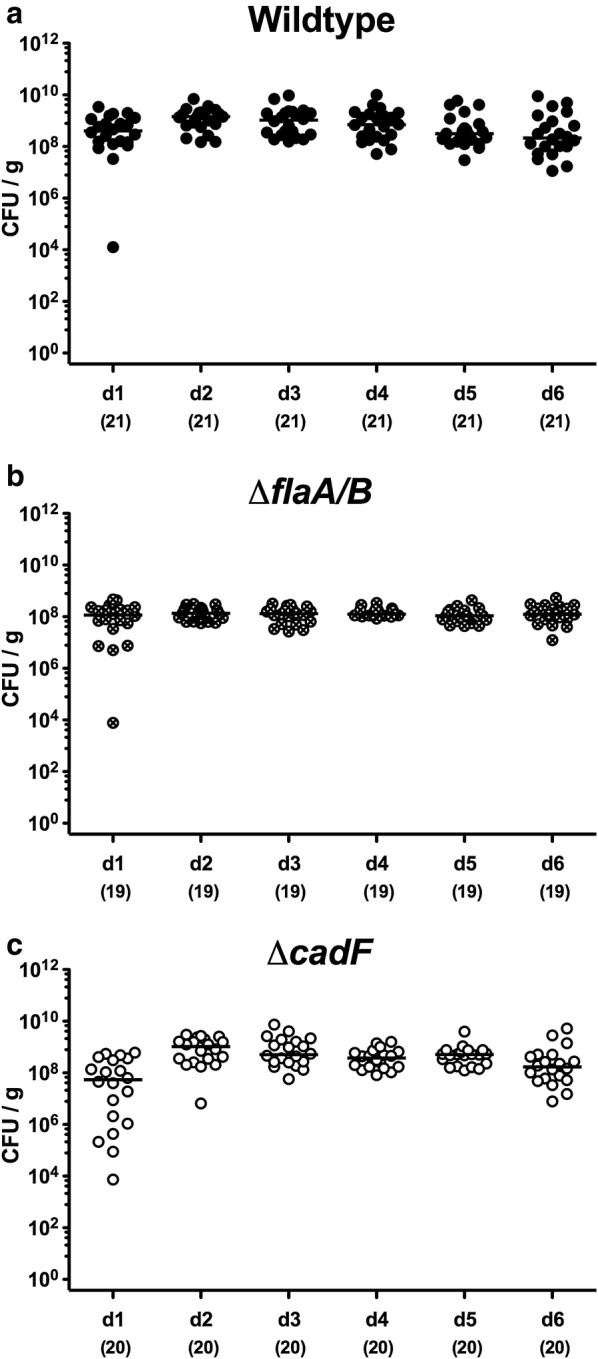
Fecal shedding of *C. jejuni* over time following peroral infection of secondary abiotic IL-10^−/−^ mice. On days 0 and 1, the mice were perorally challenged with **a** *C. jejuni* 81-176 WT (closed circles, here and in all other figures), **b** the isogenic mutant *ΔflaA/B* (crossed circles) or **c** the isogenic mutant *ΔcadF* (open circles). Individual fecal bacterial loads were surveyed over 6 days post-infection by culture and expressed as CFU/g. Medians (black bars) and numbers of analyzed mice (in parentheses) are indicated, and data were pooled from four independent experiments (here and in all other figures)

**Fig. 2:**
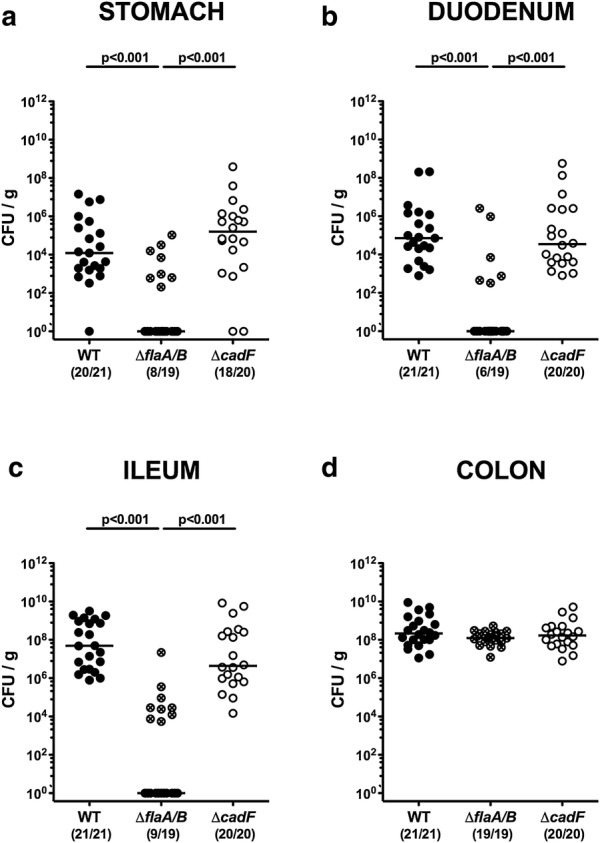
Gastrointestinal loads of WT, Δ*flaA/B* and Δ*cadF C. jejuni* at day 6 post-infection. Bacterial loads were determined in **a** the stomach, **b** the duodenum, **c** the ileum, and **d** the colon at day 6 post-infection following challenge with 81-176 WT (closed circles), *ΔflaA/B* (crossed circles) or *ΔcadF* (open circles)

We first determined whether inactivation of *flaA/B* or *cadF* had an impact on gastrointestinal colonization of *C. jejuni* in the secondary abiotic IL-10^−/−^ mice model, by comparing these mutants with isogenic WT bacteria. Approximately 10^9^ viable bacteria of each strain were orally fed on days 0 and 1. As early as 24 h following the first infection and until the end of the observation period (i.e., day 6 p.i.), median fecal pathogenic loads of up to 10^9^ colony-forming units per g (CFU/g) were determined for all three tested bacterial strains (Fig. 1). At day 6 p.i., the animals were sacrificed and *C. jejuni* content of the complete gastrointestinal tract was quantified by culture. As expected, the highest loads were present in the ileum and colon, while stomach and duodenum contained approximately four log lower counts (Fig. 2). The numbers of the Δ*flaA/B* mutant were lower in luminal samples taken from the stomach, duodenum, and ileum, compared to the other two strains (p < 0.001; Fig. 2a–c), but no difference was found in the colon (Fig. 2d). Hence, inactivation of *flaA/B*, but not of *cadF*, leads to a compromised colonization potential of *C. jejuni* in the proximal gastrointestinal tract, while colonization of the distal part of the gastrointestinal tract is not affected by these gene deficiencies.

### Clinical impact of *C. jejuni* motility and adhesion in infected secondary abiotic IL-10^−/−^ mice

**Fig. 3:**
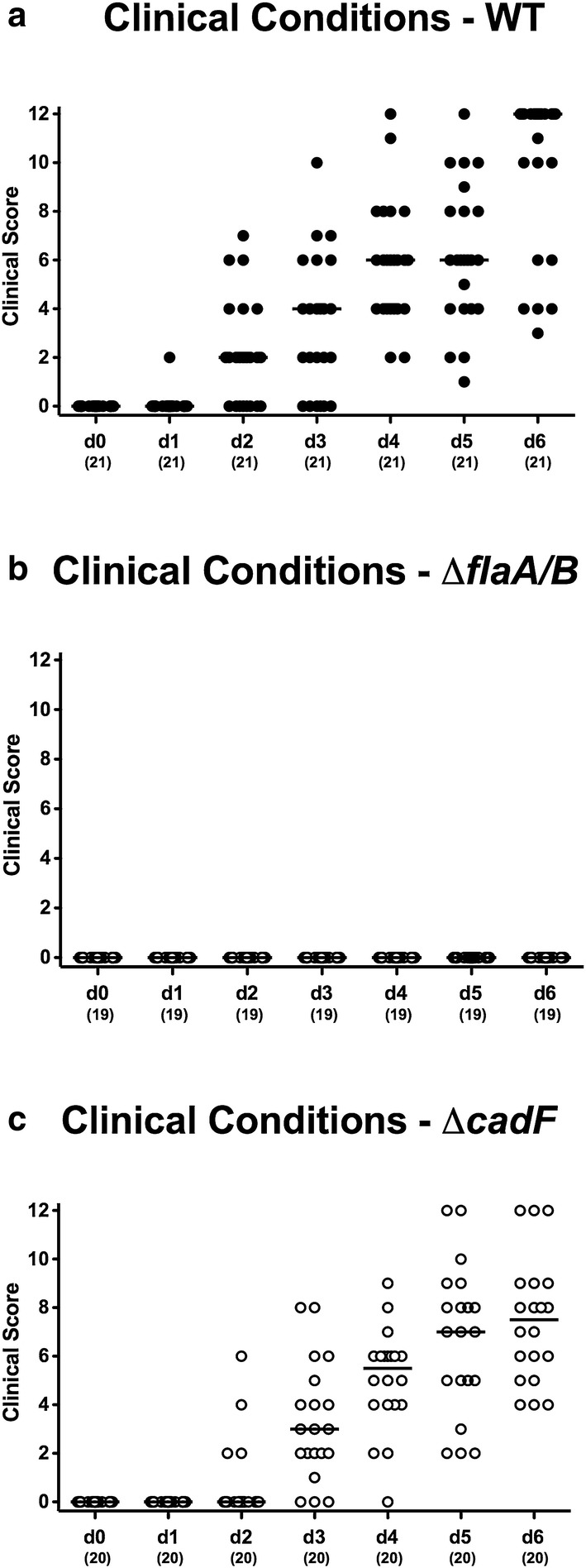
Clinical conditions over time of mice challenged with the three *C. jejuni* strains. Clinical conditions were monitored during the 6 days post challenge with **a** WT, **b** *ΔflaA/B*, and **c** *ΔcadF*, and these were quantitatively assessed applying a standardized clinical scoring system

**Fig. 4:**
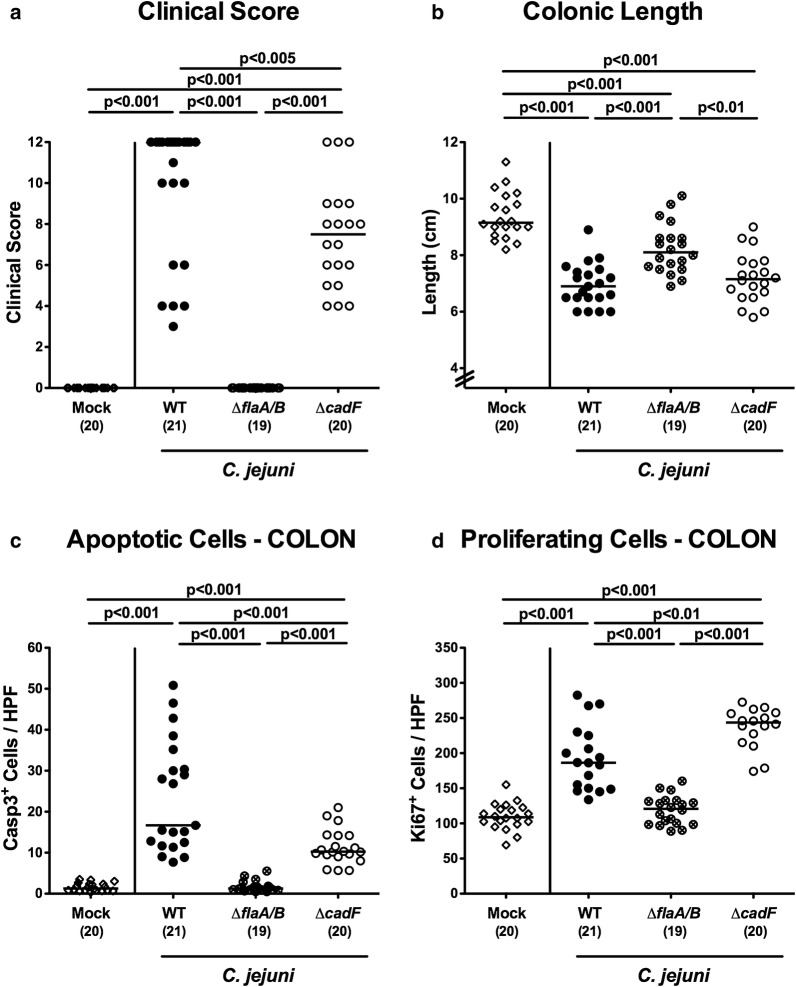
Macroscopic and microscopic parameters at day 6 post-infection. Macroscopic *C. jejuni* induced sequalae determined at day 6 included **a** clinical conditions and **b** colonic length. Microscopic intestinal changes were quantitated by the average numbers of **c** colonic epithelial apoptotic cells (positive for caspase-3, Casp3), and **d** of proliferating/regenerating cells (positive for Ki67) from six high power fields (HPF, ×400 magnification) per animal in immunohistochemically stained colonic paraffin sections at day 6 post-infection. Mock challenged mice (open diamonds) served as negative controls

We next addressed whether comparable colonic loads of the respective *C. jejuni* strains were associated with similar pathogen-induced disease outcomes. Whereas mice displayed increasing clinical scores starting at day 2 following infection with WT and Δ*cadF* bacteria, indicative for progressive *C. jejuni*-induced disease (Fig. 3a, c), infection with the Δ*flaA/B* mutant left the mice clinically uncompromised (Fig. 3b). In fact, by day 6 p.i., mice colonized with the WT strain were suffering from severe signs of campylobacteriosis including wasting and bloody diarrhea, while none of the animals infected with Δ*flaA/B* exerted symptoms, similar to mock infected control mice (p < 0.001; Fig. 4a); the Δ*cadF* infected mice, however, displayed slightly lower clinical scores as compared to WT strain infected counterparts (p < 0.005; Fig. 4a). Hence, despite high intestinal pathogenic loads, murine infection with the Δ*flaA/B* mutant did not result in overt campylobacteriosis, whereas inactivation of the *cadF* gene only marginally impaired the ability to cause symptoms.

### Relevance of *C. jejuni* motility and adhesion in induction of intestinal apoptosis and epithelial regeneration

Given that intestinal inflammation is accompanied by shortening of the affected intestinal compartment [28, 33], we measured the colonic lengths upon necropsy. Irrespective of the applied strain, *C. jejuni* infected mice exhibited shorter large intestines as compared to mock control animals (p < 0.001; Fig. 4b). The effect was weaker for animals infected with the Δ*flaA/B* mutant, whose colonic lengths were longer compared to parental WT or Δ*cadF* challenge (p < 0.001 and p < 0.01, respectively; Fig. 4b).

We further addressed whether the lack of apparent symptoms upon Δ*flaA/B* infection could be corroborated microscopically. Given that apoptosis is regarded as a reliable marker for the grading of intestinal inflammatory conditions [31], we quantitatively assessed caspase3 + colonic epithelial cell responses. The colonic samples from mice infected with Δ*cadF* and WT contained significantly increased numbers of apoptotic cells (p < 0.001 vs naive), whereas slightly lower cells were apoptotic in the colonic epithelia following Δ*cadF* infection compared to WT (p < 0.001; Fig. 4c; Additional file 1: Fig. S1A); notably, no increase was observed following Δ*flaA/B* infection. Furthermore, the numbers of Ki67 + cells, indicative for cell proliferation and regeneration, had increased considerably in the colonic epithelia of mice infected with Δ*cadF* or WT bacteria (p < 0.001 vs naive), whereas these cell numbers did not differ between Δ*flaA/B* infected and mock infected control mice (Fig. 4d; Additional file 1: Fig. S1B). Hence, in contrast to peroral challenge with WT and the Δ*cadF* mutant, infection with non-motile *C. jejuni* Δ*flaA/B* did neither result in significant macroscopic nor microscopic inflammatory sequelae. These observations make it unlikely that presence of the bacteria in the stomach and duodenum were solely due to coprophagy.

### *C. jejuni* motility and adhesion in induction of colonic immune cell responses

**Fig. 5:**
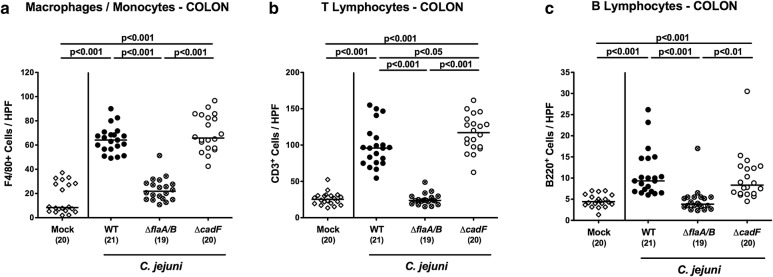
Immune cell responses in the large intestine. The average numbers of immune cells were determined microscopically from six HPF (×400 magnification) per infected or control animal using immunohistochemically stained colonic paraffin sections. Shown are data for **a** macrophages and monocytes (F4/80+), **b** T lymphocytes (CD3+), and **c** B lymphocytes (B220+)

The three *C. jejuni* strains were also compared for their ability to elicit innate and adaptive immune cell responses within the large intestines of infected mice. Peroral infection with the WT and Δ*cadF*, but not the Δ*flaA/B* strain was associated with a marked increase in innate immune cell subsets, such as F4/80 + macrophages and monocytes in the colonic mucosa and lamina propria (p < 0.001; Fig. 5a; Additional file 1: Fig. S1C). Adaptive immune cells such as CD3 + T lymphocytes and B220 + B lymphocytes had all increased in the large intestinal mucosa and lamina propria in the case of WT and Δ*cadF* (p < 0.001), but this was not observed during Δ*flaA/B* infection (Fig. 5b, c; Additional file 1: Fig. S1D, E). Interestingly, colonic T cell numbers were even slightly higher in animals that had received Δ*cadF* compared to WT (p < 0.05; Fig. 5b, c; Additional file 1: Fig. S1D). Hence, murine infection with the *C. jejuni* Δ*cadF* mutant and its parental strain, but not with the Δ*flaA/B* mutant, resulted in pronounced innate and adaptive immune cell responses in the large intestines.

### *C. jejuni* motility and adhesion in intestinal pro-inflammatory mediator secretion

**Fig. 6:**
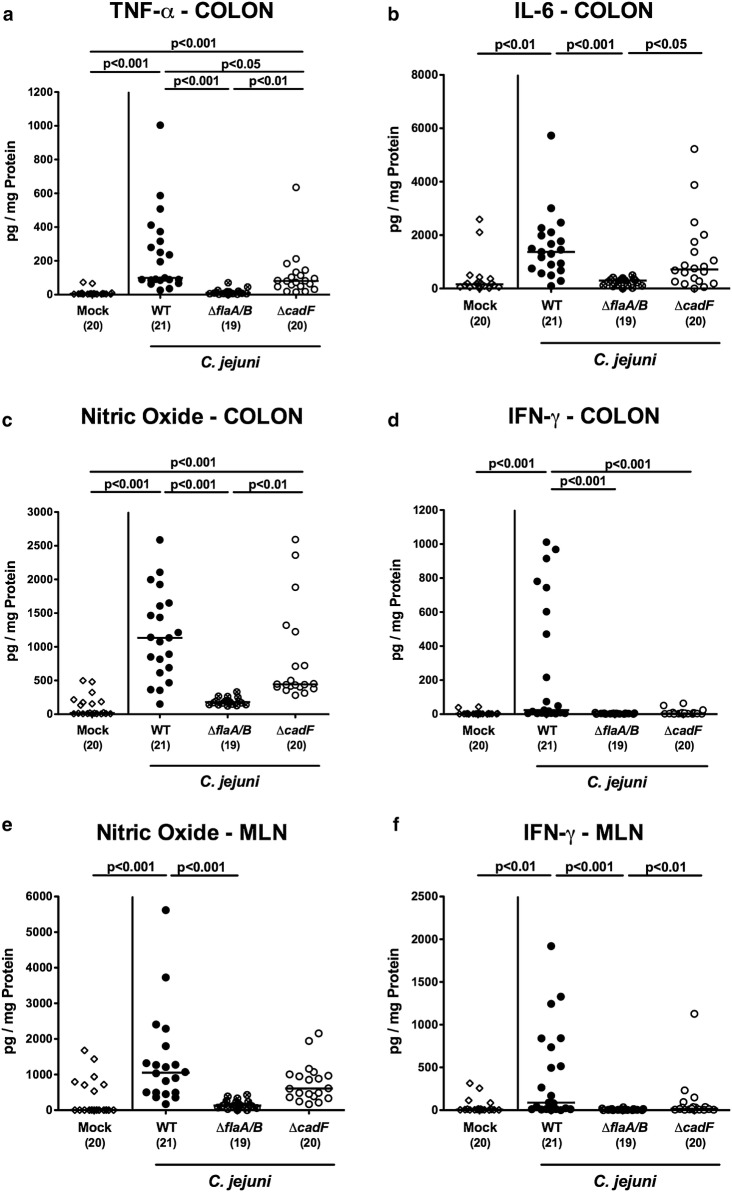
Intestinal pro-inflammatory mediator responses. Levels were determined for colonic **a** TNF-α, **b** IL-6, **c** nitric oxide, and **d** IFN-γ and for **e** nitric oxide and **f** IFN-γ in supernatants of ex vivo biopsies derived from mesenteric lymph nodes (MLN)

We next measured pro-inflammatory mediators in distinct parts of the intestinal tract following *C. jejuni* infection. Colonic secretion of TNF-α and nitric oxide was increased exclusively upon infection with WT and Δ*cadF* strains (p < 0.001; Fig. 6a, c). Following WT strain infection only, colonic levels of IL-6 and IFN-γ were elevated (p < 0.01 and p < 0.001, respectively; Fig. 6b, d), which also held true for nitric oxide and IFN-γ concentrations in ex vivo biopsies derived from mesenteric lymph nodes (MLN) at day 6 p.i. (p < 0.001 and p < 0.01, respectively; Fig. 6e, f).

Hence, murine infection with Δ*cadF*, but not Δ*flaA/B* deficient bacteria resulted in enhanced pro-inflammatory mediator secretion in the intestinal tract.

### *C. jejuni* motility and adhesion in extra-intestinal and systemic pro-inflammatory immune responses

**Fig. 7:**
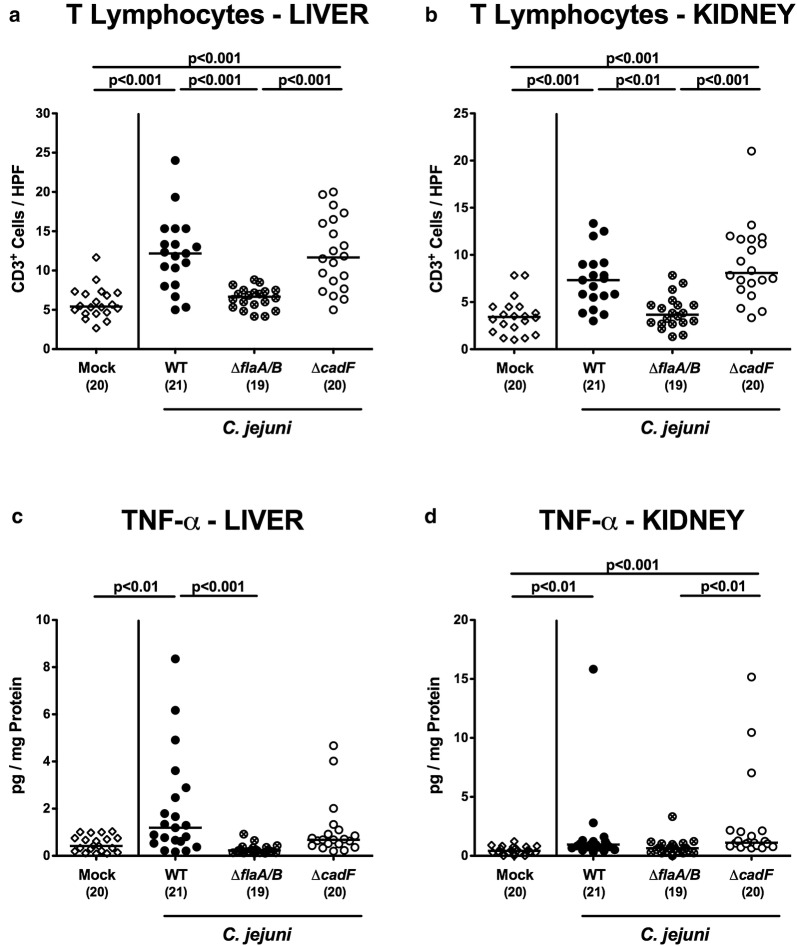
Pro-inflammatory immune responses in extra-intestinal tissues. Data are shown for T lymphocytes (CD3+) (**a**, **b**) and for TNF-α concentrations (**c**, **d**) in liver (**a**, **c**) and kidneys (**b**, **d**) of infected and control animals

**Fig. 8:**
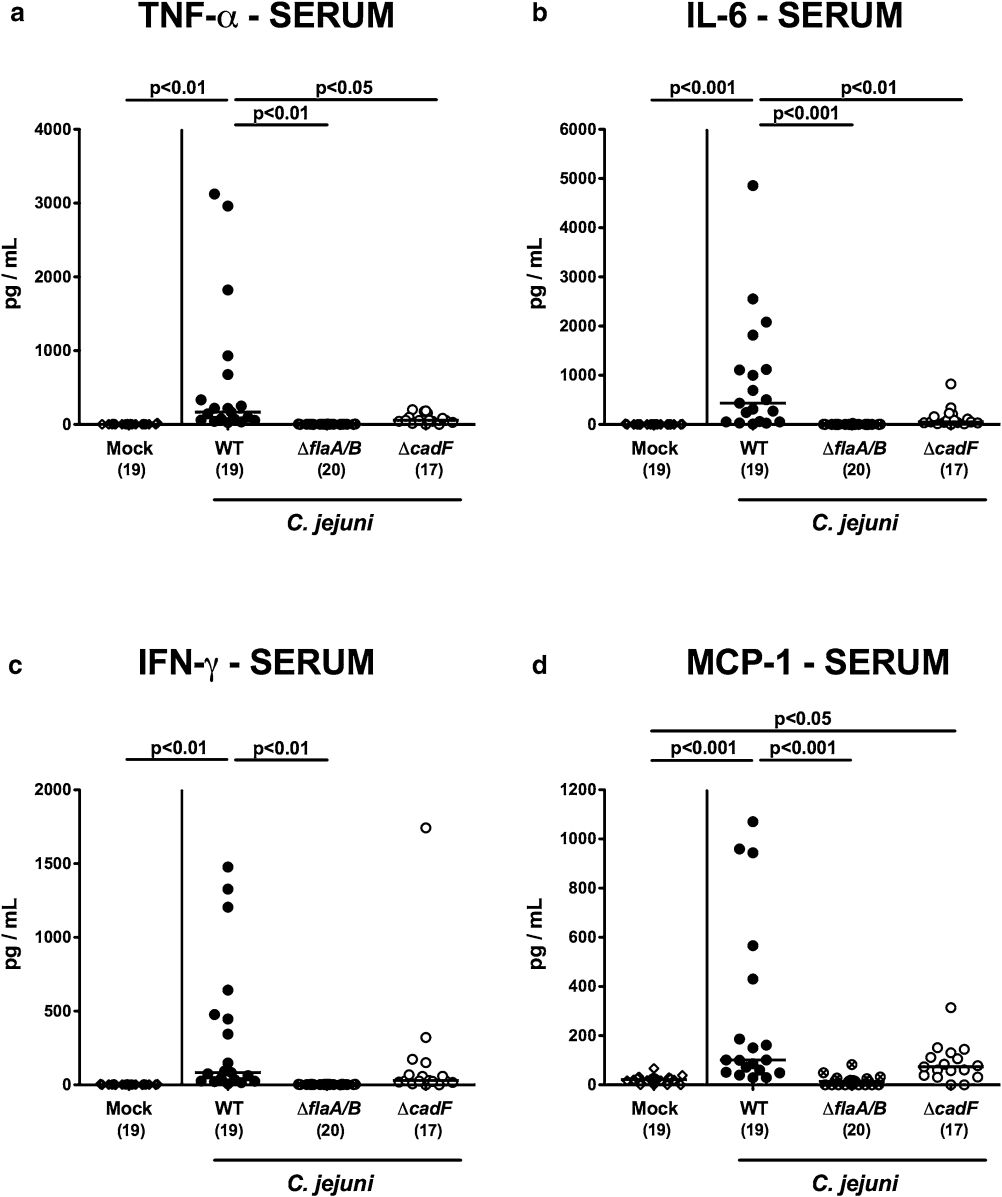
Systemic pro-inflammatory mediator responses following *C. jejuni* infection. Data are shown for **a** TNF-α, **b** IL-6, **c** IFN-γ, and **d** MCP-1 in serum samples taken 6 days post-infection

We next assessed whether the immunopathological differences observed between the *C. jejuni cadF* and *flaA/B* mutants were extended to extra-intestinal and even systemic compartments. For extra-intestinal sites, we determined numbers of CD3 + T cells and TNF-α secretion in liver and kidneys. Infection with WT or Δ*cadF* but not Δ*flaA/B* resulted in elevated numbers of T lymphocytes in either organ (p < 0.001; Fig. 7a, b), and in slightly increased TNF-α secretion in the kidneys (p < 0.01–0.001; Fig. 7d). In the liver, however, only WT strain infection was associated with elevated TNF-α concentrations (p < 0.01; Fig. 8c). Furthermore, strain-dependent differences in immunopathological responses upon *C. jejuni* infection could also be observed systemically: Mice infected with the WT, but not the Δ*flaA/B* strain produced increased systemic levels of TNF-α, IL-6, IFN-γ, and MCP-1 (p < 0.001; Fig. 8), whereas in Δ*cadF* infected mice, elevated MCP-1 serum concentrations could be measured (p < 0.05 vs mock; Fig. 8d).

**Fig. 9:**
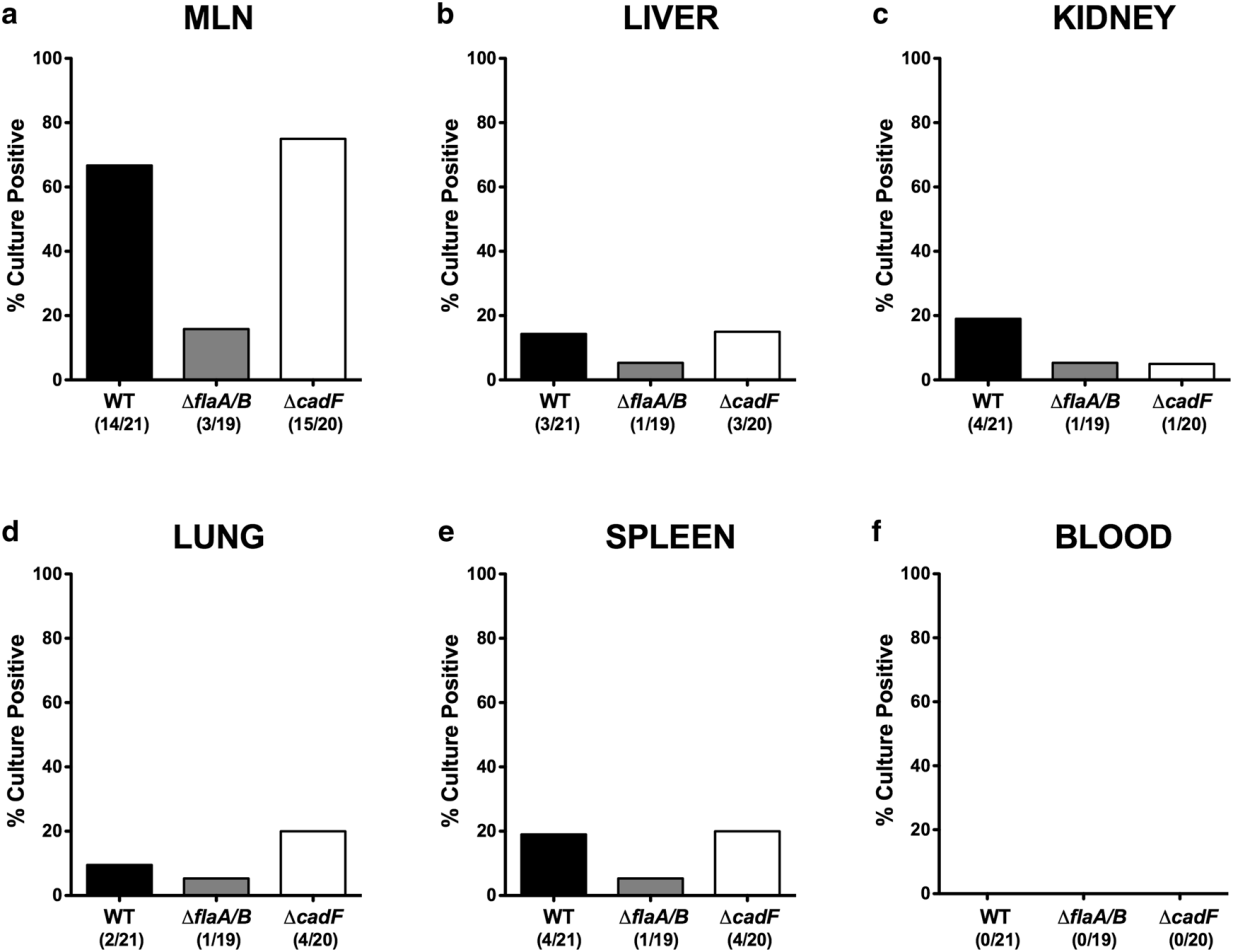
Bacterial loads of extra-intestinal organs as a result of bacterial translocation. The bacterial loads were quantitatively assessed in ex vivo biopsies at day 6 p.i. derived from **a** MLN, **b** liver, **c** kidneys, **d** lungs, **e** spleen, and **f** cardiac blood by culture. The cumulative relative translocation rates of viable bacteria in each tissue out of four independent experiments are presented as %

We finally addressed whether the observed differences in extra-intestinal and systemic pro-inflammatory responses could be due to different levels of translocated bacteria. Samples of various organs were cultured for presence of *C. jejuni*, which revealed their presence in MLN, liver, lungs, and spleen in a number of animals, though all cardiac blood cultures were negative (Fig. 9). The relative abundance of viable bacteria was lower in MLN, liver, lungs, and spleen of animals infected with Δ*flaA/B* compared to the other two strains, while culture of kidney homogenates resulted in fewer positive samples for the two mutants compared to WT. These data indicate that murine infection with *C. jejuni* WT or the *cadF* mutant was accompanied with marked extra-intestinal and even systemic pro-inflammatory immune responses, that were absent in case of Δ*flaA/B*, and that these were paralleled by detectable amounts of viable organisms in various tissue sites. The lower numbers of Δ*flaA/B* bacteria in extra-intestinal organs suggests that these non-motile bacteria were less able to translocate from the gut to other tissues.

## Discussion

Both the bacterial flagella and the adhesin CadF are well-investigated pathogenicity and virulence factors of *C. jejuni*, respectively, and are considered key players for colonization and subsequent host cell invasion [2–4]. In vitro studies revealed that CadF-mediated invasion of intestinal epithelial cells represents a crucial prerequisite for *C. jejuni* to initiate immunopathological responses via induction of cytokine responses [19, 21, 22, 34–36]. In fact, these immunopathological sequelae of infection help to explain the severity of symptoms during acute campylobacteriosis which are induced by cells of the innate immune system [37]. In our present study, we provide in vivo evidence that FlaA/B and CadF exert differential features in the interaction of *C. jejuni* and the mammalian host. By means of our clinical murine infection model, we show that *C. jejuni* flagellar motility but not adhesion exerted by CadF is required for induced immunopathology in the murine host. Neither inactivation of the flagellin genes nor of the *cadF* gene resulted in a compromized large intestinal colonization by *C. jejuni* as indicated by comparably high colonic loads of either bacterial strain. However, whereas WT bacteria and the *cadF* deficient mutant could also be isolated from the stomach, duodenum and ileum upon peroral infection, these sites were only poorly colonized by the non-motile mutant strain. This pronounced phenotype provides strong evidence that motility is required to allow *C. jejuni* to escape the unfavorable luminal conditions exerted by acids, bicarbonate and lytic enzymes within the lumen of the upper environmental tract, for instance. In this scenario motility allows the bacteria to reach mucus sites where the pathogen is protected from toxic influences and can adhere to epithelial cells to prevent passive transport to the colon where even the non-motile bacteria accumulate due to the low peristaltics.

It is well established that flagella-deficient *C. jejuni* mutants are unable to colonize the gastrointestinal tract of infant mice [38, 39], of young chicks [40] and of piglets [41]. However, a role of CadF in colonisation of the mammalian gut was mostly inferred from in vitro data, as it was shown previously that *cadF*-deficient *C. jejuni* mutants poorly adhered to cultured mammalian cells [21, 25, 34], though CadF was reported to be essential for cecal colonization in chicken [26]. Our data provide strong evidence, however, that CadF is not essential for murine colonization, and only marginally affects the outcome of infection. Thus, one or more different adhesin(s) are obviously required for *C. jejuni* colonization and disease development in mice. Because this might reflect the situation in humans, it will be important to identify these factors in further screens.

The difference between mutants deficient in flagellins or *cadF* extended beyond colonization capacity, given that there were also noted differences in their ability to generate macroscopic disease signs and microscopic inflammatory responses in the large intestines. Interestingly, WT and *cadF* deficient bacteria were able to enhance pro-inflammatory mediator secretion in distinct compartments of the intestinal tract, which was not seen with the non-motile *C. jejuni* mutant lacking flagella. The murine model applied here also allowed to investigate the capacity to generate campylobacteriosis-like symptoms in mice. Surprisingly, high numbers of *C. jejuni* present in the colon were not per se responsible for triggering disease, as could be demonstrated with the Δ*flaA/B* mutant that colonized the colon effectively, but did not cause enteric disease. In a recent study applying a murine *C. jejuni* induced enteritis model, however, mice could not be infected by a *flaA*-deficient mutant strain and did therefore not display any signs of enteritis [42]. Notably, in this study the gut microbiota of corresponding mice was not completely eradicated and this leads to the assumption that the residual microbiota established after vancomycin treatment is responsible for the complete colonization defect of the *flaA* deficient mutant. This provides evidence that motility is required to allow *C. jejuni* to escape from commensal bacteria that produce harmful metabolites and thus create an unfavorable environment for the pathogen.

In our study, the presence of symptoms coincided with the ability to generate intestinal, extra-intestinal and systemic immune responses, which both WT bacteria and the Δ*cadF* mutant were capable of. This observation further confirms the hypothesis of an immunopathological nature of campylobacteriosis. It has been hypothesized that bacterial invasion into host cells is regulated by pro-inflammatory mediators in the gut [43] and this idea is in line with our observations, since non-motile mutant strains, that are impaired in their capacity to invade concurrently exhibited far weaker immune responses.

A marked difference was further observed in the ability of *C. jejuni* to reach extra-intestinal sites. All three bacterial strains could be isolated from extra-intestinal organs, but the numbers of viable bacteria were much lower for the non-motile Δ*flaA/B* mutant. As expected, it appeared that due to lack of flagella-dependent motility, fewer bacteria were able to reach liver, lungs, and spleen. Alternatively, it has been shown that the flagellum of *C. jejuni* can act as a type III secretion apparatus for the delivery of bacterial factors such as the Cia or Fed proteins into the extracellular milieu or directly into host cells in vitro [18–20]. Thus, certain exported *C. jejuni* proteins may also trigger the above responses in mice. Thus, future studies should be designed to provide evidence for one or both of these options.

## Conclusion

For the first time, our presented in vivo data provide evidence that *C. jejuni* FlaA/B, but not CadF are pivotally involved in inducing campylobacteriosis upon peroral infection of the vertebrate host. Future studies should unravel the underlying mechanisms of the host–pathogen interactions in more detail. Furthermore, the here applied clinical murine infection model of secondary abiotic IL-10^−/−^ mice has been proven suitable not only for detailed investigations of immunological aspects of campylobacteriosis, but also for differential analyses of the roles of distinct *C. jejuni* virulence factors in induction and progression of disease.

## Methods

### Ethics approval

All animal experiments were conducted in accordance with the European Guidelines for animal welfare (2010/63/EU) following approval of the protocol by the commission for animal experiments headed by the “Landesamt für Gesundheit und Soziales” (LaGeSo, Berlin, registration number G0247/16). Clinical conditions of mice were surveyed twice daily.

### Generation of secondary abiotic mice and *C. jejuni* infection

IL-10^−/−^ mice of C57BL/6j background were reared and housed under specific pathogen free conditions. In order to counteract physiological colonization resistance and hence facilitate intestinal pathogenic colonization, secondary abiotic mice with a virtually depleted gut microbiota were generated upon broad-spectrum antibiotic treatment as reported earlier [31, 33].

Sex-matched, 3 months old mice were perorally infected with either the *C. jejuni* parental strain 81-176 (WT), the isogenic *flaA/B* deletion mutant (Δ*flaA/B*), or the *cadF* deletion mutant (Δ*cadF*). An inoculum of 10^9^ CFU in 0.3 mL phosphate buffered saline (PBS; Gibco, life technologies, UK) was administered on two consecutive days (i.e., days 0 and 1) by oral gavage. Mock control animals received an equal volume PBS perorally. Mice were maintained in a sterile environment and had unlimited access to autoclaved food and drinking water and were handled under strict aseptic conditions to avoid contamination.

### Monitoring of clinical conditions

The clinical conditions of the mice were surveyed prior and post respective *C. jejuni* infections on a daily basis by applying a standardized cumulative clinical score (maximum 12 points). These scores included the abundance of blood in feces as detected by the Guajac method using a Haemoccult, Beckman Coulter (PCD, Krefeld, Germany) (score 0: no blood; 2: microscopic detection of blood; 4: macroscopic blood visible), presence of diarrhea (score 0: formed feces; 2: pasty feces; 4: liquid feces), and by visual clinical and behavioral symptoms (score 0: normal; 2: ruffled fur and/or less locomotion; 4: isolation, severely compromised locomotion, pre-final aspect) as described earlier [29].

### Sampling procedures

At day 6 post-infection (p.i.), the animals were sacrificed upon isoflurane inhalation (Abbott, Germany). Luminal gastrointestinal samples from stomach, duodenum, ileum and colon, and ex vivo biopsies from colon, ileum, mesenteric lymph nodes (MLN), liver, kidneys, lungs, and spleen were taken under sterile conditions. Intestinal samples were collected from each mouse in parallel for microbiological, immunohistopathological and immunological analyses. The absolute colonic lengths were measured with a ruler (in cm).

### Immunohistochemistry

In situ immunohistochemical analyses were performed in colonic ex vivo biopsies that had been immediately fixed in 5% formalin and embedded in paraffin as described earlier [44–46]. Paraffin sections (5 μm) of ex vivo biopsies from colon, liver and kidneys were stained with primary antibodies directed against cleaved caspase 3 (Asp175, Cell Signaling, Beverly, MA, USA, 1:200) for detection of apoptotic epithelial cells; against Ki67 (TEC3, Dako, Denmark, 1:100) for detection of proliferating epithelial cells; against F4/80 (# 14-4801, clone BM8, eBioscience, San Diego, CA, USA, 1:50) for detection of macrophages/monocytes; against CD3 (#N1580, Dako, 1:10) for detection of T lymphocytes; and against B220 (No. 14-0452-81, eBioscience; 1:200) for detection of B lymphocytes. Secondary antibodies were used for detection as previously described [31, 47]. Positively stained cells were examined by light microscopy (magnification 100× and 400×), and for each mouse the average number of respective positively stained cells was determined within at least six high power fields (HPF, 0.287 mm^2^, 400× magnification) by an independent investigator using blinded samples.

### Bacterial colonization

The number of viable *C. jejuni* bacteria was quantitatively assessed in feces over time p.i., in homogenates of ex vivo biopsies taken MLN, spleen, liver, kidneys and lungs, and in cardiac blood at day 6 p.i. by culture as described elsewhere [31, 47]. The detection limit of viable bacteria was ≈ 100 CFU per g.

### Pro-inflammatory mediator detection in supernatants of intestinal and extra-intestinal ex vivo biopsies

Colonic ex vivo biopsies were cut longitudinally, washed in PBS, and strips of approximately 1 cm^2^ tissue as well as ex vivo biopsies derived from MLN (3 lymph nodes), liver (approximately 1 cm^3^), one kidney (cut longitudinally), and one lung were placed in 24-flat-bottom well culture plates (Nunc, Germany) containing 500 μL serum-free RPMI 1640 medium (Gibco, life technologies, UK) supplemented with penicillin (100 U/mL) and streptomycin (100 µg/mL; PAA Laboratories, Germany). After 18 h at 37 °C, culture supernatants were tested for tumor necrosis factor- (TNF-) α, interleukin (IL)-6, interferon (IFN)-γ, and monocyte chemoattractant protein (MCP)-1 by the Mouse Inflammation Cytometric Bead Array (CBA; BD Biosciences, Germany) on a BD FACSCanto II flow cytometer (BD Biosciences). Nitric oxide was measured by the Griess reaction as reported previously [33]. Systemic pro-inflammatory mediator concentrations were assessed in serum samples.

### Statistical analysis

Medians and levels of significance were determined by one-way ANOVA test followed by Tukey post-correction for multiple comparisons (GraphPad Prism v7, USA). Two-sided probability (p) values ≤ 0.05 were considered significant. Experiments were reproduced three times and pooled data are shown.

## Additional file

**Additional file 1: Figure S1.** Representative photomicrographs illustrating apoptotic and proliferating colonic epithelial as well as immune cells responses in large intestinal and extra-intestinal compertments in secondary abiotic IL-10^−/−^ mice following peroral *flaA/B* or *cadF* gene deficient *C. jejuni* infection. Secondary abiotic IL-10^−/−^ mice were perorally challenged either with the *C. jejuni* 81-176 wildtype strain (WT), the isogenic *flaA/B* gene deletion mutant (*ΔflaA/B*) or the isogenic *cadF* gene deletion mutant (*ΔcadF*) by gavage on days 0 and 1. Mock mice served as negative controls. Photomicrographs reepresentative for four independent experiments illustrate (A) apoptotic colonic epithelial cells (Casp3+), (B) proliferating colonic epithelial cells, large intestinal (C) macrophages and monocytes (F4/80+), (D) T lymphocytes (CD3+), (E) B lymphocytes (B220+) and furthermore, (F) hepatic and (G) renal T lymphocytes (CD3+) in at least six high power fields (HPF) as quantitatively assessed in respective paraffin sections applying *in situ* immunohistochemistry at day 6 post-infection (100× magnification, scale bar 100 μm).

## Data Availability

Please contact author for data requests.

## Abbreviations

cadF: *Campylobacter* adhesin to fibronectin
CBA: Cytometric Bead Array
CFU: colony-forming units
FlaA/B: flagella filament A/B
HPF: high power field
IFN: interferon
IL: interleukin
MAPK/ERK: mitogen-activated protein kinases/extracellular signal-regulated kinases
MCP: monocyte chemoattractant protein
MLN: mesenteric lymph nodes
n.s.: not significant
NF-κB: nuclear factor kappa-light-chain-enhancer of activated B cells
Nod: nucleotide-oligomerization-domain
PBS: phosphate-buffered saline
p.i.: post-infection
TLR: Toll-like receptor
TNF: tumor necrosis factor
WT: wildtype
