# Supplementary material for: Immunopathological properties of the Campylobacter jejuni flagellins and the adhesin CadF as assessed in a clinical murine infection model
Source: Gut Pathog. 2019 May 17;11:24. doi: 10.1186/s13099-019-0306-9 (PMC6525468; doi:10.1186/s13099-019-0306-9)

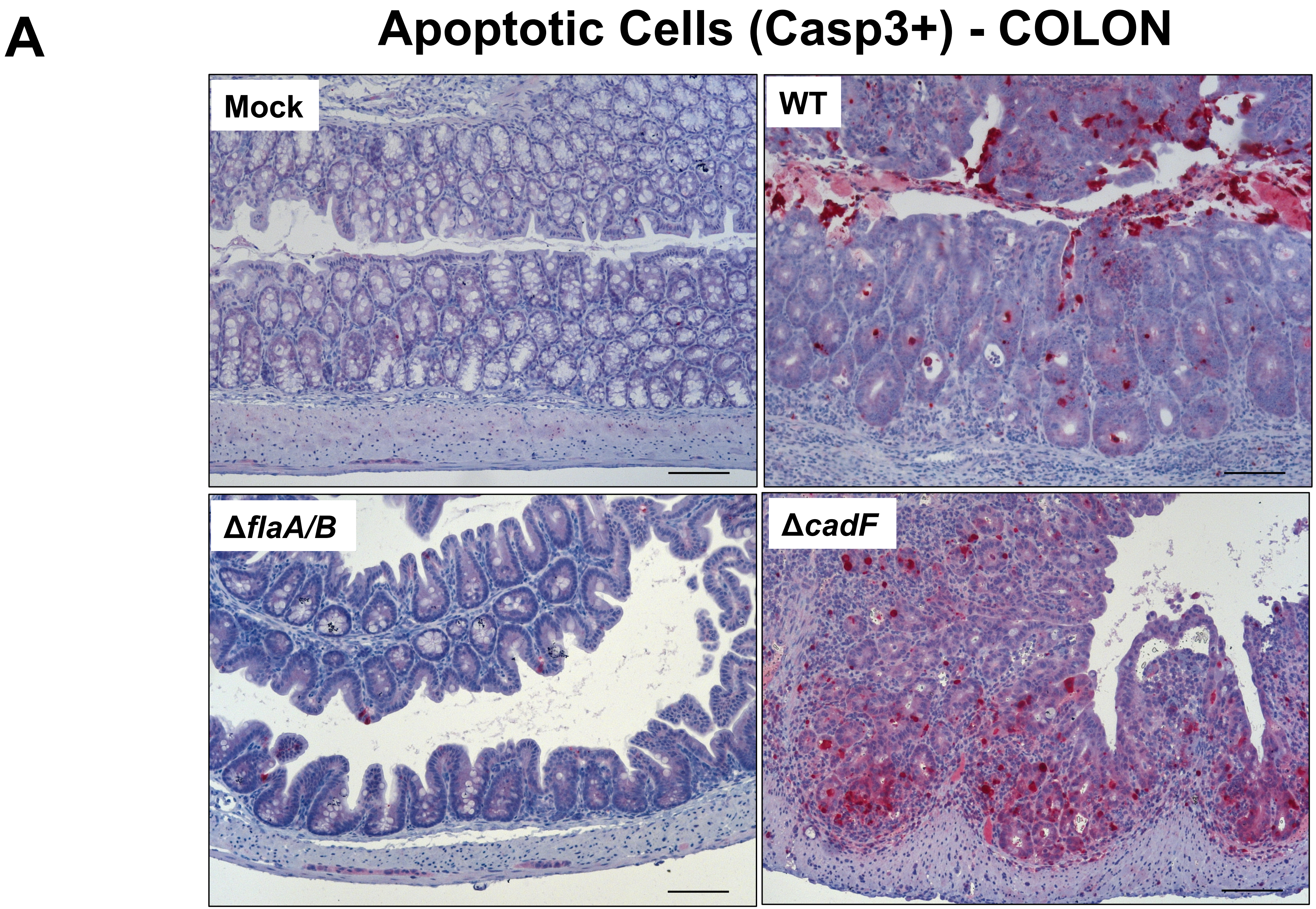
A
Apoptotic Cells (Casp3+) - COLON
Mock
WT
ΔflaA/B
ΔcadF

B

Proliferating Cells (Ki67+) - COLON

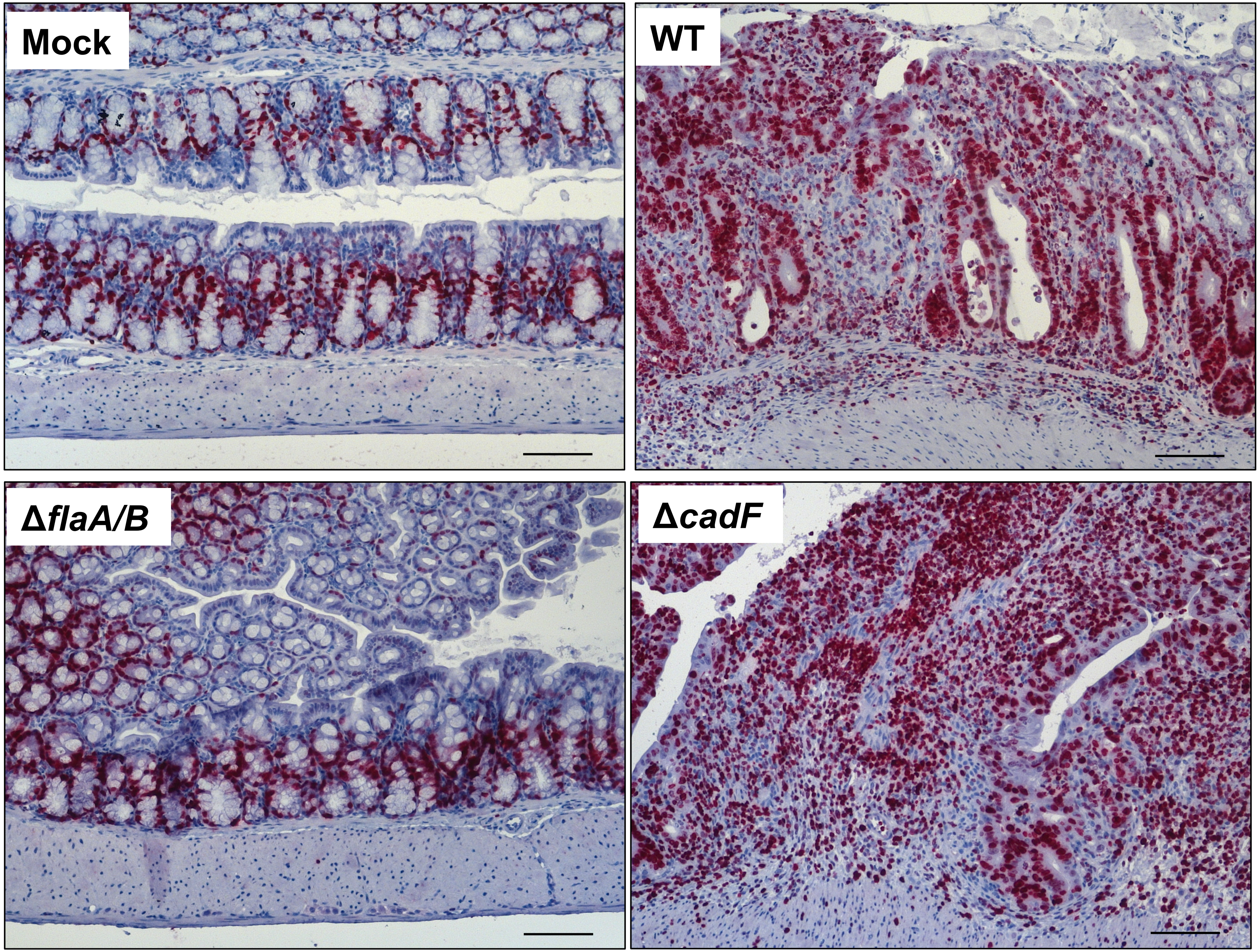

C

Macrophages / Monocytes (F4/80+) - COLON

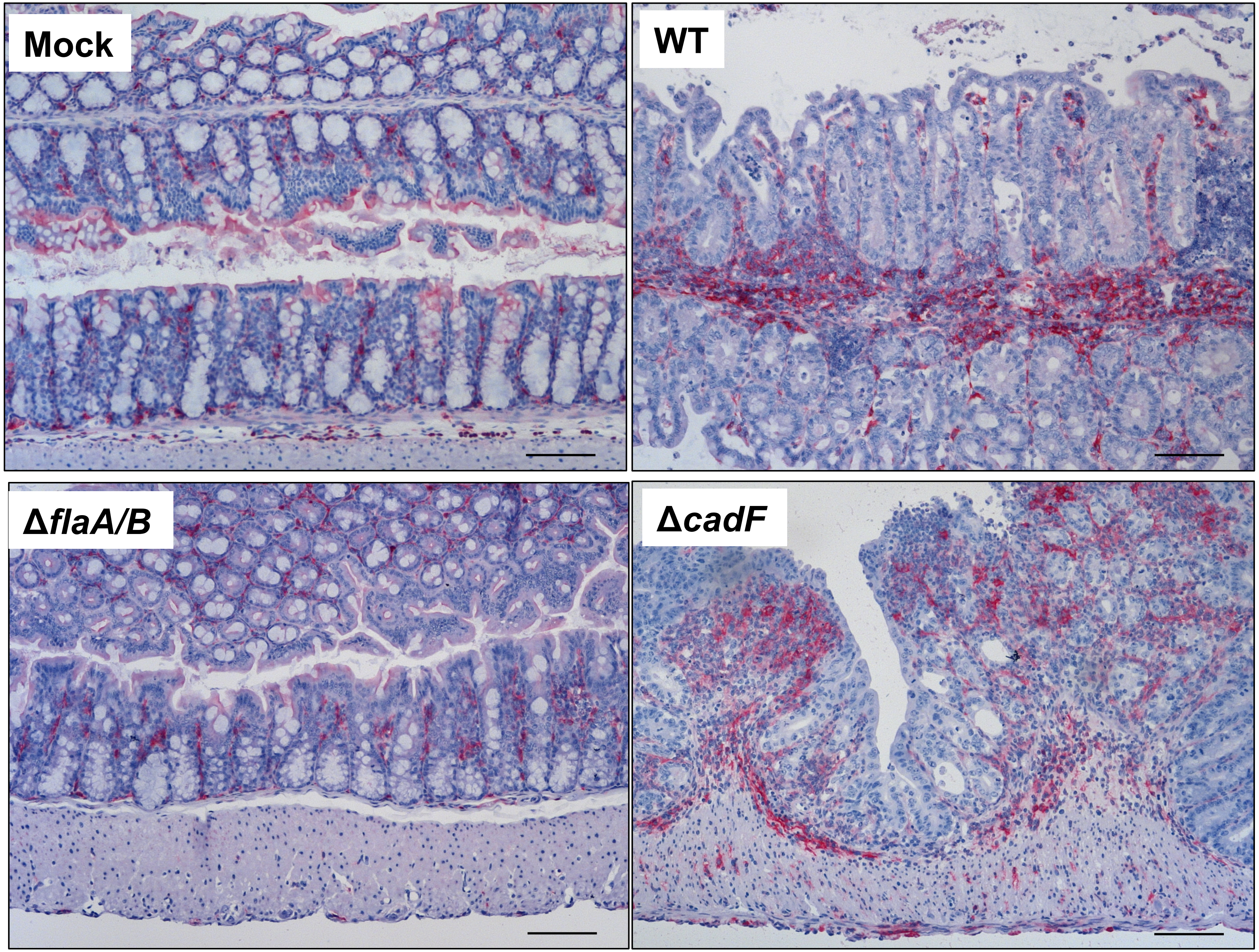

D

T Lymphocytes (CD3+) - COLON

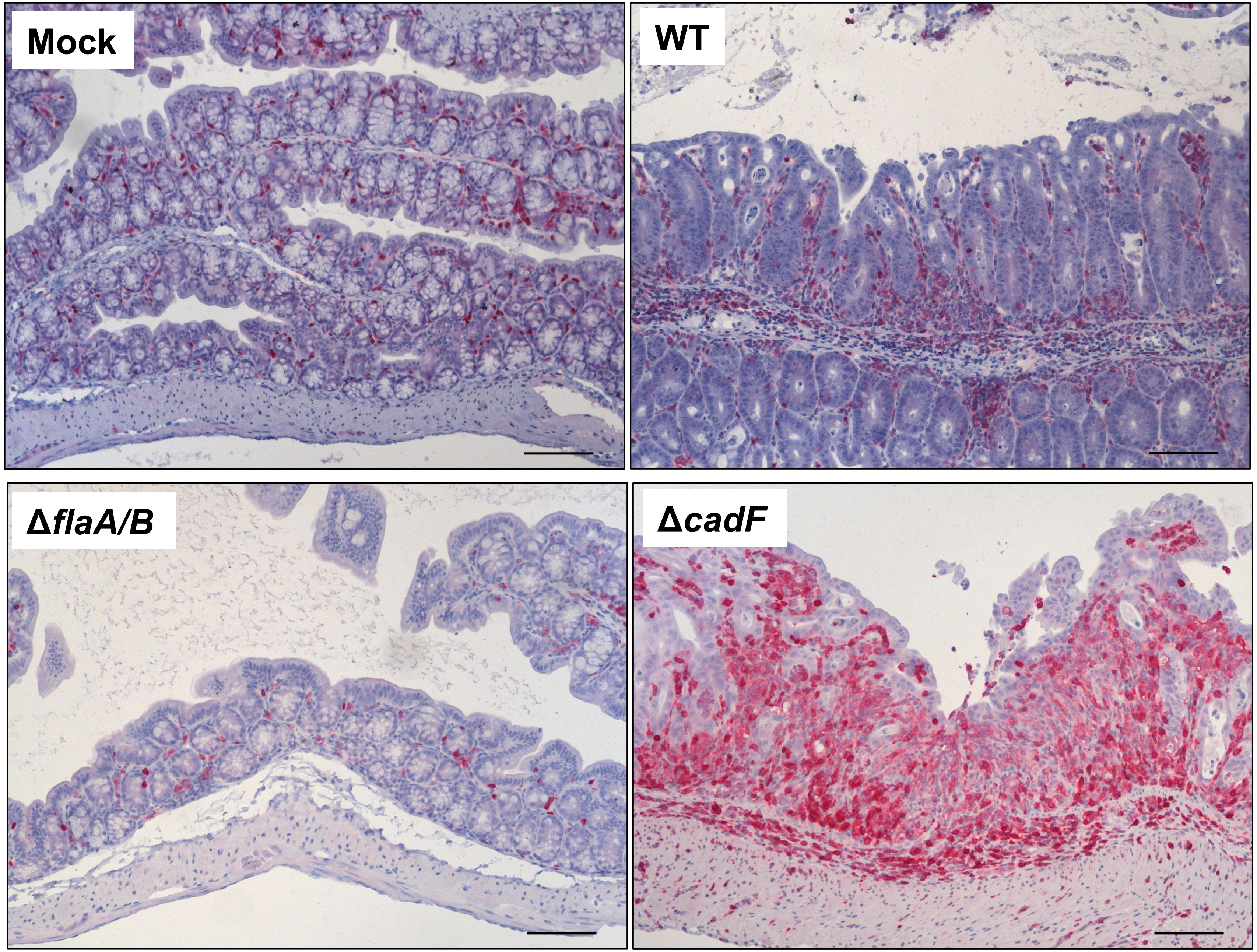

E

B Lymphocytes (B220+) - COLON

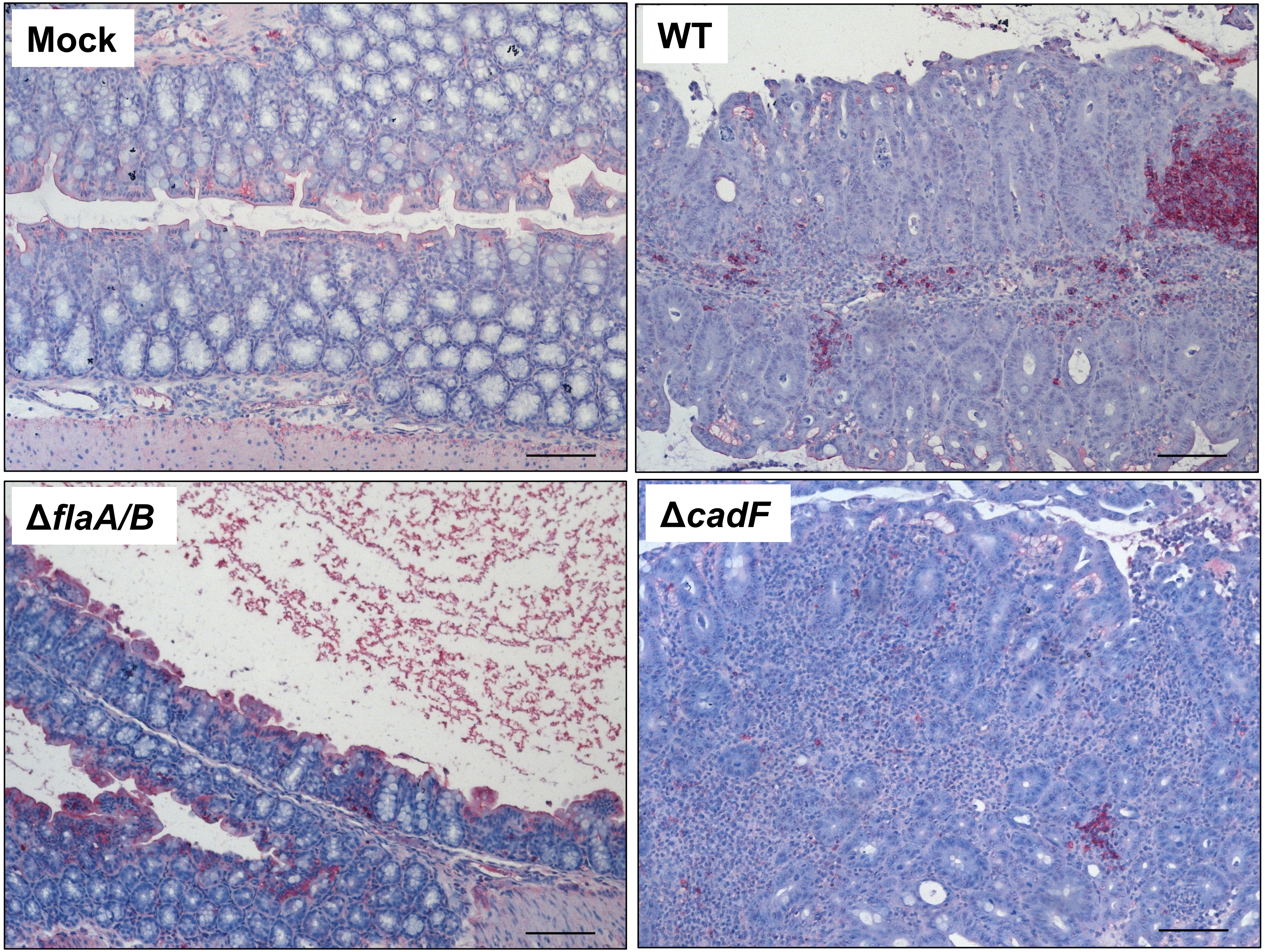

F

T Lymphocytes (CD3+) - LIVER

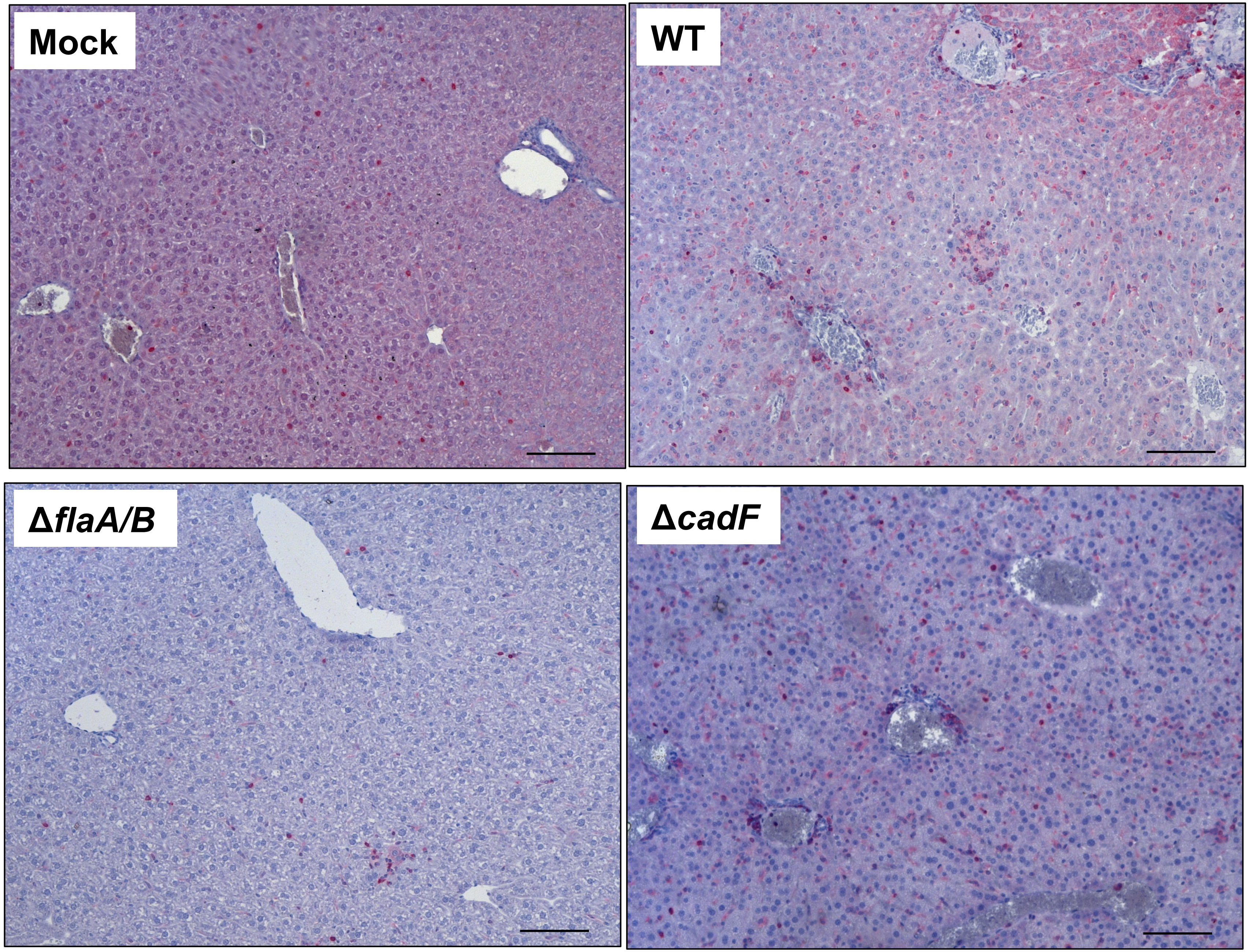

G

T Lymphocytes (CD3+) - KIDNEY

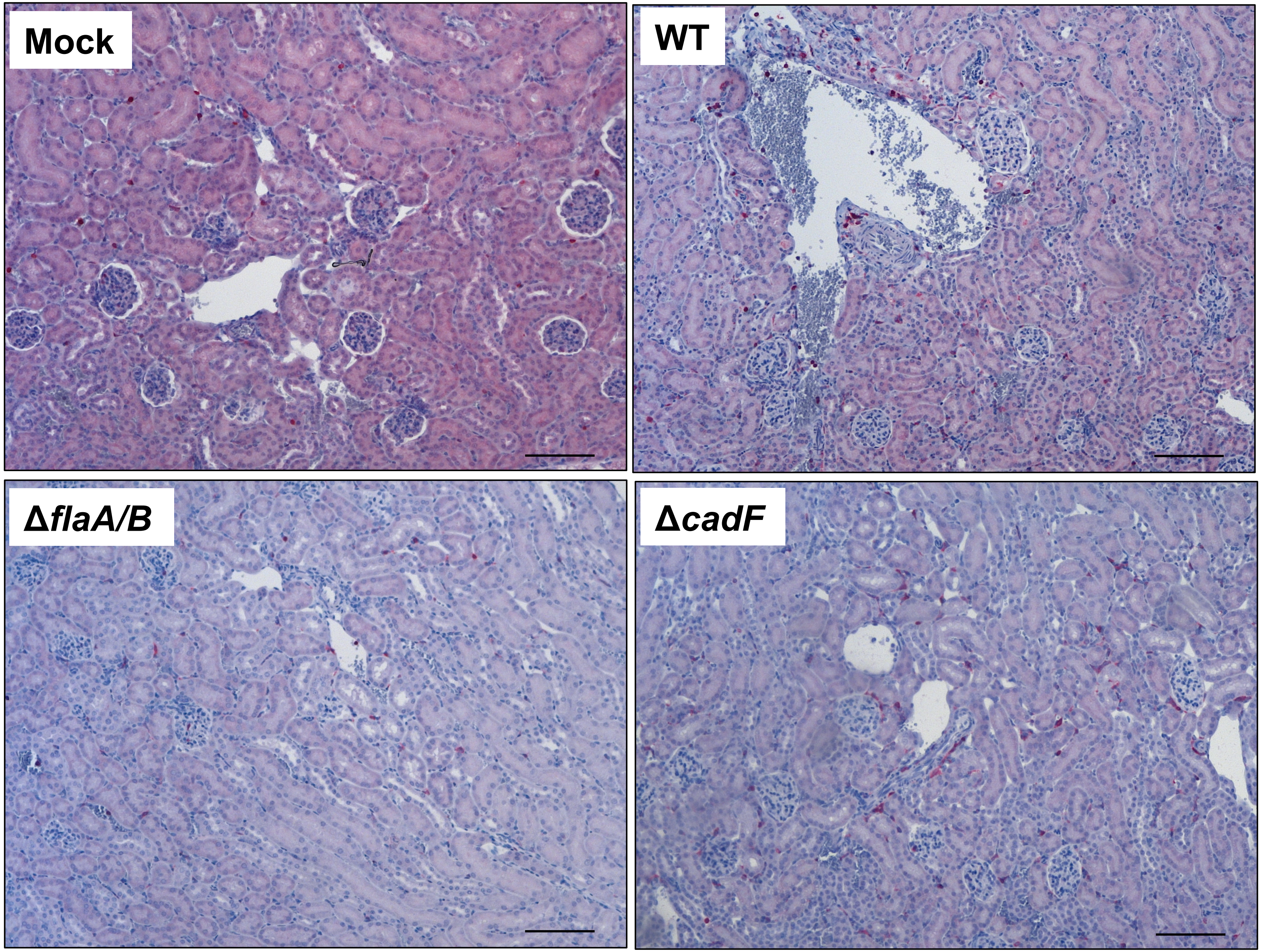

Supplement: Supplementary file 1 — Additional file 1: Figure S1. Representative photomicrographs illustrating apoptotic and proliferating colonic epithelial as well as immune cells responses in large intestinal and extra-intestinal compertments in secondary abiotic IL-10−/− mice following peroral flaA/B or cadF gene deficient C. jejuni infection. Secondary abiotic IL-10−/− mice were perorally challenged either with the C. jejuni 81-176 wildtype strain (WT), the isogenic flaA/B gene deletion mutant (ΔflaA/B) or the isogenic cadF gene deletion mutant (ΔcadF) by gavage on days 0 and 1. Mock mice served as negative controls. Photomicrographs reepresentative for four independent experiments illustrate (A) apoptotic colonic epithelial cells (Casp3+), (B) proliferating colonic epithelial cells, large intestinal (C) macrophages and monocytes (F4/80+), (D) T lymphocytes (CD3+), (E) B lymphocytes (B220+) and furthermore, (F) hepatic and (G) renal T lymphocytes (CD3+) in at least six high power fields (HPF) as quantitatively assessed in respective paraffin sections applying in situ immunohistochemistry at day 6 post-infection (100× magnification, scale bar 100 μm). [file 13099_2019_306_MOESM1_ESM.pdf]
